# Evaluation of Serum Levels of Transient Receptor Potential Cation Channel Subfamily V Member 1, Vasoactive Intestinal Polypeptide, and Pituitary Adenylate Cyclase-Activating Polypeptide in Chronic and Episodic Migraine: The Possible Role in Migraine Transformation

**DOI:** 10.3389/fneur.2021.770980

**Published:** 2021-12-23

**Authors:** Mansoureh Togha, Zeinab Ghorbani, Samira Ramazi, Fahime Zavvari, Fariba Karimzadeh

**Affiliations:** ^1^Headache Department, Iranian Center of Neurological Research, Neuroscience Institute, Tehran University of Medical Sciences, Tehran, Iran; ^2^Cardiovascular Diseases Research Center, Department of Cardiology, School of Medicine, Heshmat Hospital, Guilan University of Medical Sciences, Rasht, Iran; ^3^Department of Clinical Nutrition, School of Medicine, Guilan University of Medical Sciences, Rasht, Iran; ^4^Department of Physiology, Medical School, Iran University of Medical Sciences, Tehran, Iran; ^5^Cellular and Molecular Research Center, Iran University of Medical Sciences, Tehran, Iran

**Keywords:** TRPV1, VIP, PACAP, migraine, migraine transformation

## Abstract

**Objectives:** This study aimed to investigate the role of serum levels of transient receptor potential cation channel subfamily V member 1 (TRPV1), vasoacive intestinal peptide (VIP), and pituitary adenylate cyclase-activating polypeptide (PACAP) in the development and also the transformation of migraine in patients suffering from migraine.

**Methods:** Eighty-nine participants with a mean age of 39 years were divided into 23 episodic migraine (EM), 36 chronic migraine (CM), and 30 healthy control groups. Demographic, anthropometric, and headache characteristic information, and also blood samples, was collected. Serum levels of TRPV1, VIP, and PACAP were measured using the enzyme-linked immunosorbent assay (ELISA) technique.

**Results:** Based on our findings, the serum level of TRPV1 was significantly higher in CM compared to the control group (*p* < 0.05), whereas serum levels of VIP (*p* < 0.01) and PACAP (*p* < 0.05) in the EM group were significantly more than the control group. There was no significant difference between EM and CM groups.

**Conclusions:** An elevation in the serum levels of TRVP1 among chronic migraineurs and increments in the levels of VIP and PACAP were observed among EM patients compared to healthy subjects. However, our data failed to demonstrate the probable role of these biomarkers in migraine progression, and more studies are needed to clarify the molecular mechanisms involved in migraine progression.

## Introduction

Migraine is a prevalent debilitating neurological disorder with moderate to severe headache which lasts 4 or 72 h. Headache is often unilateral with associating symptoms of photophobia, phonophobia, nausea, or vomiting (1). In one-third of patients, the aura (transient focal neurological symptoms) precedes the headache. Migraine is divided into two types of chronic (CM) and episodic (EM) based on the number of headaches occurring monthly. EM can change to CM, which is much more severe and characterized by headaches that exceed more than 14 days per month with at least 3 months of repetition (2, 3).

Migraine imposes a heavy socioeconomic burden on society and this is while its exact mechanism is not yet fully known (2, 4–9). Genetics, environmental factors, metabolic changes, and hormones, may all contribute to the onset of migraines. A number of well-studied mechanisms that are probably involved in migraine pathogenesis are as follows: trigeminovascular pain pathway, proinflammatory cytokines, and neuroinflammation, and also the activity of some factors such as nitric oxide and neuropeptides including calcitonin gene-related peptide (CGRP), substance P, neurokinin A, neuropeptide Y (NPY), vasoactive intestinal peptide (VIP), and pituitary adenylate cyclase-activating polypeptide (PACAP) (5–9).

Among these neuropeptides, VIP, a 28-amino acid peptide, is a vasodilator that releases from the cranial parasympathetic preganglionic and cerebral perivascular nerves. Although VIP plasma levels were shown to increase after migraine attacks and/or interictal period in both EM or CM, its infusion did not induce migraine attack. It has been proposed that VIP may play a role in triggering migraine chronification through vasodilation and nociceptor sensitization (10, 11). Besides, PACAP is a peptide that is mainly made up of 38 amino acids and is also found in the sensory ganglion, parasympathetic ganglion, and secondary neurons of the trigeminal nucleus. This neuropeptide seems to be similar to VIP structurally and could cause vasodilation. Moreover, PACAP could increase trigeminal nociceptor's excitability by increasing cAMP. The infusion of this neuropeptide was shown to stimulate headache in migraineurs. In this regard, targeting the inhibition of PACAP receptors has been investigated for migraine treatment (12–15). PACAP levels were observed to be elevated during migraine headaches and found to be decreased by sumatriptan, a medication used in the treatment of migraine (16, 17). Additionally, transient receptor potential vanilloid 1 (TRPV1), a Na^+^ and Ca^2+^ permeable channel, is among the other factors which are assumed to play a role in migraine pathogenesis. TRPV1 could be activated by capsaicin, severe heat, low pH, inflammatory factors, and some lipid-derived substances such as anandamide (7, 8, 18–20). This channel is found in trigeminal ganglions and its stimulation might cause the release of other neuropeptides such as CGRP. Therefore, the role of TRPV1 in the pathophysiology of migraine has attracted much attention in the recent years. It should be noted that the agonists and antagonists of this channel are under investigation for therapeutic aspects in migraine disease (7, 8, 19, 20).

Considering the unknown molecular mechanism involved in migraine pathogenesis and the possible role of various agents in the progression of EM to CM, this study aimed to investigate the plasma levels of some less-studied factors including VIP, PACAP, and TRPV1 in patients suffering from EM, CM, and healthy controls.

## Materials and Methods

### Study Population

In this case-control study, the population was comprised of 89 subjects (71 women and 18 men) with an average age of 39 years who were divided into three groups including subjects with chronic migraine (CM group, *n* = 36 patients), individuals with episodic migraine (EM group, *n* = 23 patients), and healthy subjects (control group, *n* = 30 headache-free volunteers). Based on the convenience sampling method, the sampling process was performed from September 2017 to June 2020 at Sina University Hospital Headache Clinic, Tehran University of Medical Sciences, Tehran, Iran. Following an advertisement using posters describing the study aims placed all over the hospital (primarily in headache clinic), the patients with migraine and the age- and sex-matched non-headache controls, who were healthy subjects from the hospital staff or patient companions, were included in this study. Diagnosis of EM and CM was performed by a neurologist based on the third edition of International Headache Society criteria (ICHD-III) (21).

The inclusion criteria considered for enrolling in this study were as follows: age range between 18 and 65, having a body mass index (BMI) between 18.5 and 35 kg/m^2^, not being pregnant or breastfeeding, and not having a positive medical history for any of the following disorders: cardiovascular, infectious, or endocrinological diseases, renal, hepatic, immunological, and allergic disorders, and also other chronic neurological diseases such as Alzheimer's disease, multiple sclerosis, epilepsy, or Parkinson's disease. Besides, having migraine headaches (with or without aura) for at least 6 months prior to the study and excluding the diagnosis of medication overuse headache (MOH) were the specific inclusion criteria for the case group. Subjects who did not meet the mentioned conditions or were unwilling to fill out a questionnaire were excluded. The study protocol was approved by National Institute for Medical Research Development (NIMAD) (grant number 957537) and confirmed by the ethical committee of NIMAD with ID: IR.NIMAD.REC.1396.054. After a complete explanation of the research process, all participants filled out the consent forms.

### Demographic, Anthropometric, and Clinical Information of Patients

After the initial interview and demographic data collection, anthropometric measurements were performed based on the method provided by the World Health Organization. Height and weight were measured to calculate BMI that was obtained as weight (kg) divided by height squared (m^2^). For the purpose of body weight measurement, Seca Clara 803 digital scale (accuracy of 0.01 gr; Seca GmbH & Co. KG., Hamburg, Germany) was used. Height was also measured using a Seca 216 wall-mount stadiometer (accurate to 0.1 cm without shoes; Seca GmbH & Co. KG., Hamburg, Germany) in bare feet. The patients were also questioned about the number and type of abortive or analgesic medication use 30 days after the first visit.

### Headache Diaries and Visual Analog Scale

In the next step, participants were visited by a neurologist or headache subspecialist (M.T.) and migraine and its type were determined based on ICHD-III criteria (2). Patients were also guided on how to fill out the headache diary form designed by senior researcher Prof. M.T. (22). These diaries were included information about the severity, duration (time elapsed from headache onset to cease of headache by itself or through abortive medications, whichever is sooner), frequency (i.e., number of headache days) and time of discontinuation of the migraine attacks, number and type of analgesics used, and the stimulating factors of headache such as menstruation and light during 30 days. Head pain severity scores were rated through the visual analog scale (VAS), a 10-cm measurement instrument; the left side (number 0) indicates the absence of pain and its right side (number 10) indicates the most severe pain.

### Blood Sample Collection and Biochemical Assessments

A 10-ml blood sample was collected from each EM participant at the second visit, about 30 days after the first visit and at least 72 h after his/her last headache attack to be more indicative of the interictal phase of migraine. For CM cases, since the headaches lasted more than 15 days (between 15 and 30 days) per month, it was not possible to collect blood samples in the interictal phase. Blood samples were divided into 18 microtubes that were stored in −80°C freezers and 10 microtubes that were kept in −20°C freezers. All serum samples were sent to the laboratory of Sina Hospital for biochemical studies. Serum levels of target factors (TRPV1, PACAP, and VIP) were then measured using commercial enzyme-linked immunosorbent assay (ELISA) kits from Bioassay Technology Laboratory (Shanghai Korain Biotech Co., Ltd, Shanghai, China) and Crystal day Biotech Co. (Shanghai Crystal day Biotech Co., Ltd., Shanghai, China). Serum levels of these biomarkers were measured as per instructions of the manufacturers of the ELISA kits. All assays were carried out in triplicate. The intraassay and interassay coefficient of variation (CV) was <8 and <10%, respectively.

### Statistical Analysis

SPSS software version 24 was used for data analysis. The normality of the data was evaluated using the Shapiro–Wilk test. All quantitative data were reported as mean [standard deviation (SD)] or median (interquartile range, IQR) and all qualitative data as percentage and frequency. Chi-squared test, independent-sample *t*-test, or Mann–Whitney *U*-test was applied for analyzing the categorical or continuous variables between the studied groups. Kruskal–Wallis and its related *post hoc t*-test were used for making comparisons between the groups. In all statistical tests, a *p*-value < 0.05 was considered significant.

## Results

### Basic Characteristics of the Studied Groups

Eighty-nine participants (71 women and 18 men) with a mean age of 39 years were divided into three groups including control (*n* = 30), CM (*n* = 36), and EM (*n* = 23). The mean (SD) of age and BMI of participants are presented in Table 1. There was no significant difference in age, gender, or BMI between the studied groups.

**Table 1:** Comparison of gender, age, and BMI between the studied groups [data are shown as means (SD)].

**Variable**	**Control (***n*** = 30)**	**Chronic migraine (***n*** = 36)**	**Episodic migraine (***n*** = 23)**	***p*** **-value**
Percentage of women	73.3	75.0	95.6	0.087
Age (years)	41 (8)	39 (8)	38 (9)	0.509
BMI	24.88 (3.70)	26.65 (4.37)	25.24 (4.38)	0.203

### Headache Characteristics

The mean of headache characteristics including frequency, duration, and severity of headache and also the use of abortive drugs were compared between episodic and CM groups. As presented in Table 2, headache frequency in the CM group was significantly higher than EM group [25.74 (5.03) vs. 8.78 (3.26), *p*-value < 0.001]. However, there was no significant difference in the duration and severity of the headache between the two groups. Moreover, the mean of abortive drug use in the CM group was significantly higher compared to the EM group [14.42 (10.25) vs. 6.00 (4.17), *p*-value < 0.001].

**Table 2:** Comparison of headache characteristics between chronic and EM groups [data are shown as means (SD)].

	**Chronic migraine** **(***n*** = 36)**	**Episodic migraine** **(***n*** = 23)**	***p*** **-value**
Headache days per month	25.74 (5.03)	8.78 (3.26)	<0.001
Headache severity (VAS)	7.42 (2.41)	7.37 (1.82)	0.936
Attack duration (hours per month)	19.33 (12.30)	15.48 (15.65)	0.296
Using abortive medication (days per month)	14.42 (10.25)	6.00 (4.17)	<0.001

### Medication Use

The medication consumption of studied subjects at baseline and after the intervention consisted of abortive [including triptans, ergotamine derivative, and non-steroidal antiinflammatory drugs (NSAIDs)] and prophylactic drugs [including propranolol, tricyclic antidepressants (TCAs), selective serotonin reuptake inhibitors (SSRIs), and serotonin–norepinephrine reuptake inhibitors (SNRIs)] were also compared between chronic and episodic migraineurs. Based on the results, there was a significant increase in NSAID intake in the EM group [*n* = 16 (69.6%)] compared to the CM [*n* = 14 (38.9%)] group. No significant differences were observed between EM and CM groups on the use of other mentioned drugs.

### Serum Concentration of TRPV1, VIP, and PACAP

The serum levels of TRPV1, VIP, and PACAP in the control, CM, and EM groups are presented in Table 3 and Figures 1A–C. The median (IQR) value of TRPV1 was higher in the CM compared to the control group [2.91 (2.72) vs. 1.61 (1.83) ng/mL, *p*-value = 0.034], but no significant differences were observed in the comparison of the EM and the control groups. Also, a comparison of serum levels of TRPV1 between the EM and the CM showed an insignificant difference. On the other hand, there was a significant increase in the median (IQR) value of VIP in the EM group when compared to the control group [303.24 (50.38) vs. 284.50 (90.40) ng/L, *p*-value = 0.027]. However, no significant differences in the serum level of VIP were found in the CM group as compared to the EM or control subjects. In addition, it was demonstrated that the median (IQR) value of PACAP in the EM group was significantly greater than that of the control group [2.72 (1.06) vs. 2.57 (0.64) ng/mL, *p-*value = 0.043]. However, PACAP elevation in the EM group was not significant compared to the CM group. Furthermore, the increment in PACAP levels in the CM group was insignificant when compared to the control group.

**Table 3:** Comparison of serum levels of TRPV1 (ng/ml), VIP (ng/l), and PACAP (ng/ml) according to the studied groups [data are shown as median (interquartile range)].

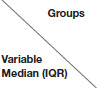	**Control** **(***n*** = 30)**	**Chronic migraine** **(***n*** = 36)**	**Episodic migraine** **(***n*** = 23)**	***p*** **-value**
TRPV1 (ng/mL)	1.61 (1.83)^#^	2.91 (2.72)^#^	1.84 (1.93)	0.034
VIP (ng/L)	284.50 (90.40)^#^	286.44 (46.83)	303.24 (50.38)^#^	0.027
PACAP (ng/mL)	2.57 (.64)^#^	2.72 (1.06)	2.73 (0.46)^#^	0.043

# *show statistically significant differences between groups*.

**Figure 1:**
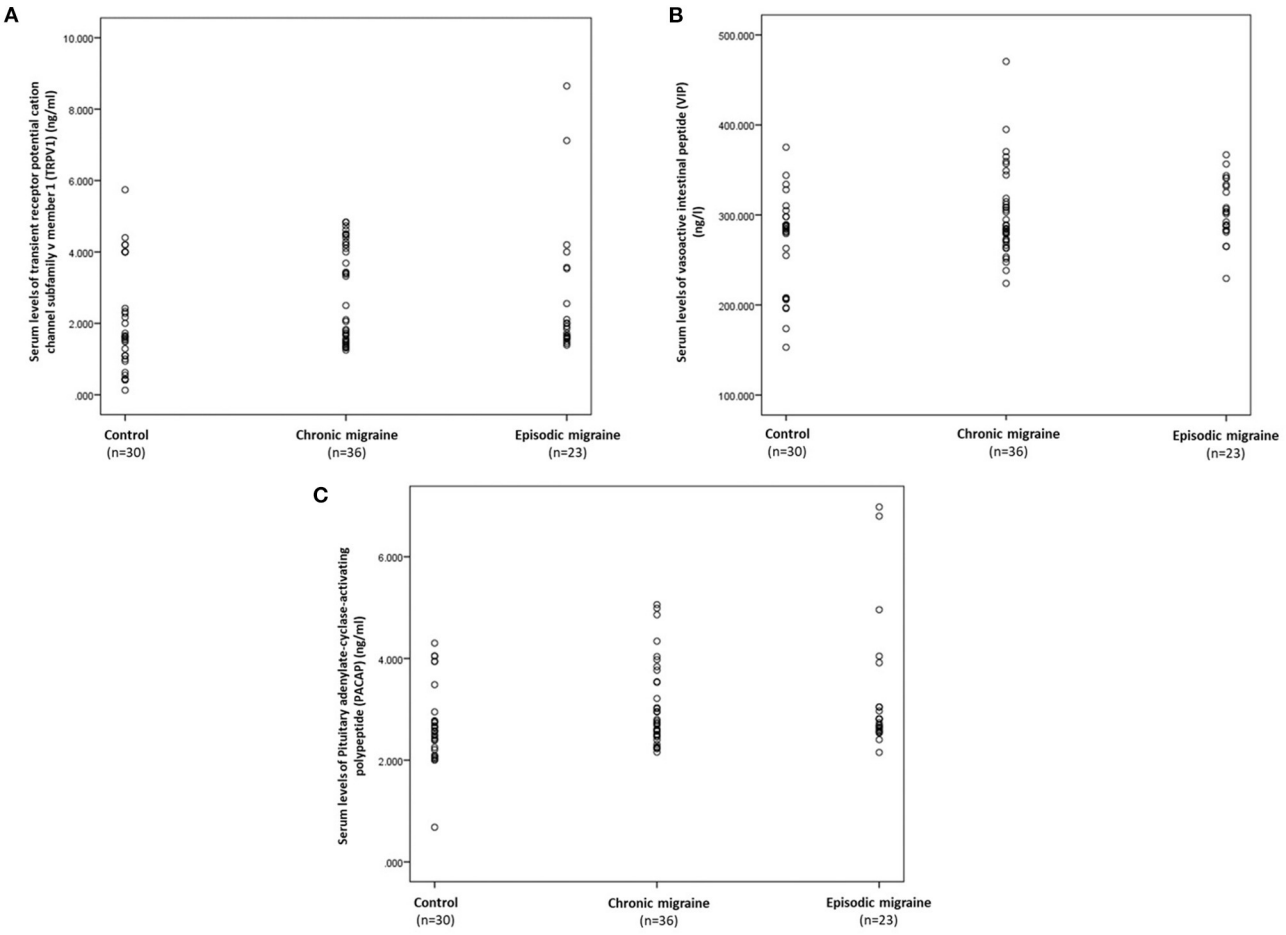
Scatter plots displaying the individual's distribution of serum concentrations of TRPV1 **(A)**, VIP **(B)**, and PACAP **(C)** according to the control, chronic, and EM groups. TRPV1, transient receptor potential vanilloid type-1 receptor; VIP, vasoactive intestinal polypeptide; PACAP, pituitary adenylate cyclase-activating polypeptide.

## Discussion

In this study, to investigate some molecular alterations involved in migraine chronification, serum levels of TRPV1, VIP, and PACAP were evaluated in patients with episodic and CM, and also healthy individuals. Based on our findings, the elevation of serum levels of VIP and PACAP was observed in patients with EMs but TRPV1 levels were higher in the serum samples of patients suffering from CMs when compared to the healthy subjects. The current findings might suggest a possible role for these factors in migraine pathogenesis, though more research is required in this area.

Unfortunately, migraine headache in 14% of episodic migraineurs can change to chronic type, which are much more severe and prolonged (23–25). So far, researchers have identified some risk factors for EM progression to a chronic form such as age, gender, obesity, and stressful life events. However, finding molecular biomarkers of the migraine patients' serum can be innovative in preventing migraine chronification (26, 27).

During the recent decades, the activation of sensory neurons in trigeminal ganglion has received more attention in the pathophysiology of migraine. TRPV1, a non-selective cation channel, is abundantly expressed in the trigeminal ganglion and its activation might lead to release of several neuropeptides involved in central sensitization including CGRP, VIP, PACAP, and substance P. These molecules are peripherally secreted from trigeminal afferents and induced intracellular elevation of cAMP or cGMP with consequent vasodilation and inflammatory events within both the dura mater and trigeminal ganglion, which is important in triggering and amplification of pain (28, 29). TRPV1 is known to exacerbate the excitability of nociceptors in response to noxious stimuli such as mechanical and thermal stimuli and proalgesic substances and therefore promote hyperalgesia. Ictal and interictal hyperalgesia, assessed using the standardized quantitative sensory testing (QST) protocol, is observed in and around the trigeminocervical region in migraine patients (30–34). These regions' sensitization seems to play a crucial role in migraine chronification, as a higher frequency of cutaneous allodynia has been observed in CM patients. A wicked circle of TRPV1 high expression and subsequently related neuropeptides overrelease could account for this phenomenon (35). TRPV1 has been suggested to have an important role in dural vasodilation, which is one of the proposed basic mechanisms in migraine pathophysiology (36, 37). Proalgesic agents can upregulate TRPV1 expression and channel activity. Ethanol has shown that may be able to induce migraines through TRPV1 stimulation followed by CGRP elevation in the trigeminovascular system (38, 39). Nitroglycerin induces CMs by increasing the mRNA expression of TRPV1 in the trigeminal ganglion (40). Capsaicin has shown that causes headache by stimulating TRPV1 and activating the extracellular signal-regulated kinase (ERK) pathway (41). Experimental studies have shown that TRPV1 antagonists could decrease the sensitization of second-order trigeminal neurons or could prevent dural vasodilation (42, 43). Sumatriptan is a migraine abortive substance through vasoconstriction and inhibition of CGRP secretion from trigeminal ganglion (44) and may also act as TRPV1 desensitizer (45, 46).

Recent research has revealed that TRPV1 single-nucleotide polymorphism may be considered as a risk biomarker of episodic to CM transformation (47). CM patients were found to have a significant TRPV1 increase in nerve fibers (mainly in C fibers) in the scalp arteries wall compared with healthy controls (48). The results of this study also showed that serum level of TRPV1 was higher in patients with CM than healthy subjects, whereas this increase was not observed in the EM group. Based on these data, it seems that serum levels of TRPV1 may have a role in migraine progression but more evaluations are needed.

Vasoactive intestinal peptide is one of the most important neuropeptides secreted from parasympathetic perivascular nerve fibers in the trigeminovascular system and acts as a potent vasodilator (49). Parasympathetic activation could be able to sensitization of afferent nociceptors, this oversensitization and repeated stimulation might have a role in the transformation of EM to the chronic one, and VIP is assumed to have a role in migraine chronification (50). Studies that conducted on people with migraine indicated that serum VIP level was elevated in CM patients with increased cranial parasympathetic system activity during migraine attacks (51) and also in the interictal period in both episodic and CM (11, 52). Cernuda-Morollón et al. have shown that interictal CGRP and VIP increased in peripheral blood in CM patients compared to healthy controls (52). Their next study showed that interictal serum VIP level was higher in CM and EM compared to healthy controls without any meaningful difference between CM and EM patients (11). Partly consistent with these prior studies, our obtained results also showed elevated serum VIP level in EM patients between headache attacks compared to the control group, but this elevation not observed in the CM patients.

Vasoactive intestinal peptide and PACAP share two common G protein-coupled receptors, VPAC_1_ and VPAC_2_, with similar affinity. PACAP has an additional specific receptor, PAC_1_, which has a higher affinity for PACAP than for VIP (53). In other words, although activation of all three receptors increases cAMP, PACAP *via* the PAC_1_ can induce adenylate cyclase activation about 100-fold more than VIP (54). So, PACAP/PAC_1_ signaling could notably elevate cAMP in peripheral trigeminal nociceptors, leading to nociception. Indeed, human and animal studies have shown that trigeminal neurons are sensitized through the elevation of cAMP (55). PACAP is a parasympathetic neuropeptide that is released from the efferent arm of the trigeminal-facial arch and has a VIP-like vasodilation property. PACAP has been proposed to have roles in mast cell degranulation, neurogenic inflammation, and migraine headaches whereas parasympathetic blocking reduces this pain (50, 56, 57). Cranial autonomic symptoms are prevalent in up to 50% of migraine patients. Likewise, these symptoms have been observed after PACAP administration (13, 58). Intravenous administration of PACAP could induce the release of CGRP in the trigeminal nucleus caudalis and lead to migraine attacks, and sumatriptan could be able to inhibit PACAP elevation (13, 16, 59). Electrical and chemical stimulation of the trigeminovascular system causes plasma PACAP elevation in rats, so it was assumed that PACAP could be considered as a biomarker in migraine pathogenesis (60). Human concordant data have also been achieved in this field. As mentioned by past results, plasma levels of PACAP were higher in both cubital and jugular veins during migraine attacks but were lower in interictal periods compared to healthy subjects (16, 61). PAC_1_ receptor blockade seems to have antimigraine effects, but more clinical trials are required to consider whether the long-term PACAP receptor blockade will have adverse side effects or not (14). Our findings showed that interictal serum PACAP levels were higher in EM patients than in the control group and PACAP increase in the CM was not enough to be significant. Our obtained results were opposite of the findings of Sara Pérez-Pereda and her colleague's research in 2020 as they showed that PACAP increases the risk of CM and not EM (10). In this regard, three points may be considered for variety of results: first, medications use, second, the time of collecting blood samples (during a migraine attack or in the interictal period), and third, parasympathetic system activity or inactivity. Mentioned factors can affect VIP and PACAP level, and also other possibly involved factors in migraine pathogenesis at the time of sampling. Therefore, considering the different results of previous studies, it seems that the role of these factors should be appraised while introducing TRPV1, VIP, and PACAP as risk biomarkers for migraine progression.

In this study, peripheral TRVP1, VIP, and PACAP were evaluated in EM patients in the interictal and in CM patients in the ictal phase. Due to the persistence of headaches more than 15 days per month (from 15 to 30 days) in CM patients, they do not have a true interictal phase of migraine and it was not possible to assess their serum biomarkers between attacks. A number of limitations can be mentioned for this study; first, as the peripheral levels of TRPV1, VIP, and PACAP were assessed merely in the interictal phase of migraine in EM patients, to achieve more comprehensive results, it is necessary to measure the CSF and serum levels of these biomarkers both between and during attacks in EM patients. Another limitation of this study was the lack of a prior sample size estimation. Moreover, applying a powered longitudinal study design, especially for exploring intraindividual longitudinal changes in these biomarkers and also the confounding factors (including medications use and comorbidities), could further clarify the associations between levels of TRPV1, VIP, and PACAP and migraine progression or reversion, which needs additional studies in the future.

## Conclusion

In conclusion, compared to healthy controls, a significant elevation in the serum levels of TRVP1 was noted among chronic migraineurs. Besides, significant increments in the levels of VIP and PACAP were observed among EM patients. These findings might be a point to investigate new strategies for antimigraine drugs. However, our data failed to demonstrate the probable role of these biomarkers in migraine progression, and more studies are needed to clarify the molecular mechanisms involved in migraine progression.

## Data Availability Statement

The original contributions presented in the study are included in the article/supplementary material, further inquiries can be directed to the corresponding authors.

## Ethics Statement

The studies involving human participants were reviewed and approved by National Institute for Medical Research Development (NIMAD) (grant no. 957537) and confirmed by the ethical committee of NIMAD with ID: IR.NIMAD.REC.1396.054. The patients/participants provided their written informed consent to participate in this study.

## Author Contributions

Content preparation, study design, acquisition, and analysis of data were all done by MT and ZG. SR, ZG, and MT drafted the manuscript. The manuscript was critically revised by MT, ZG, FK, and FZ. All authors contributed to the article and approved the submitted version.

## Funding

This research was supported by National Institute for Medical Research Development (NIMAD) (grant number 957537).

## Conflict of Interest

The authors declare that the research was conducted in the absence of any commercial or financial relationships that could be construed as a potential conflict of interest.

## Publisher's Note

All claims expressed in this article are solely those of the authors and do not necessarily represent those of their affiliated organizations, or those of the publisher, the editors and the reviewers. Any product that may be evaluated in this article, or claim that may be made by its manufacturer, is not guaranteed or endorsed by the publisher.
